# Analysis and Evaluation of Eye Behavior for Marine Operation Training - A Pilot Study

**DOI:** 10.16910/jemr.12.3.6

**Published:** 2019-12-06

**Authors:** Runze Mao, Guoyuan Li, Hans Petter Hildre, Houxiang Zhang

**Affiliations:** Norwegian University of Science and Technology, Alesund, Norway

**Keywords:** Marine operation, Eye tracking, Area of interest, Scan path, Gaze, Fixation

## Abstract

This paper presents a new analysis approach for evaluating situation awareness in marine operation training. Taking advantage of eye tracking technology, the situation awareness reflected by visual attention can be visualized and analyzed. A scanpath similarity comparison method that allows group-wise comparisons is proposed. The term ‘Expert zone’ is introduced to evaluate the performance of novice operator based on expert operators’ eye movement. It is used to evaluate performance of novice operators in groups in certain segment of marine operation. A pilot study of crane lifting experiment was carried out. Two target stages of operation for the load descending until total immersion to the seabed were selected and analyzed for both novice and expert operators. The group-wise evaluation method is proven to be able to access the performance of the operator. Besides that, from data analysis of fixation-related source and scanpath, the similarities and dissimilarities of eye behavior between novice and expert is concluded with the scanpath mode in target segment.

## Introduction

Marine operations have become more complex and diverse in recent years due to the development of technology in the marine industry. At the same time, it is reported that marine accidents such as offshore oil rig mishaps, crane mishaps and the grounding of ships, are usually followed by the development of new technologies. Human error has been established as one of the main threats to marine safety. Four-fifths of major offshore accidents are caused by human error [1]. Therefore, from safety to economy, it is important to understand what causes human error and learn from these failures. Human error has been verified to be associated with situational awareness [2]. Effectively allocating attention to maintain higher situational awareness plays a key role in detecting hazards and avoiding potential accidents. Although briefing and debriefing are introduced in marine training, evaluation of situational awareness is seldom seen. It cannot clearly and effectively reflect the potential mistake during the training and thus would lay danger in real practice.

To detect situational awareness of human, human eyes can be a reasonable option. If correctly recorded, human eyes are responsible for storing 70% of human information, along with rational analysis and visualization methods, can bring huge progress for engineering improvement. To fulfill this idea, eye tracking technology emerges at the right moment. Eye tracking, is a technique that can measure eye movements to understand the human visual focus. Because eye movements play an important part in consuming attention, the researchers invented eye trackers to objectively study the human error issue in a new way in order to prevent potential accidents. Eye tracking is implemented in many research areas with respect to training. For example, Kasarskis et al. [3], Sadasivan et al. [4], Sarter et al. [5], Yang et al. [6], Kang & Landry [7], Dehais et al. [8] and Wickens et al. [9] implemented eye tracking techniques to study flight training with a focus on fixation number, mean fixation duration, mean dwell duration and scanpath; Balk et al. [10], Fisher et al. [11], Palinko et al. [12], Paeglis et al. [13], and Xu et al. [14] studied driving training by analyzing mean fixation duration and its standard deviation, saccade size, pupil diameter and scanpath; Miall & Tchalenko [15], and Tchalenko [16] researched drawing issues introducing dwell time; Law et al. [17], and Chetwood et al. [18] carried out surgery training; Kovácsová et al. [19] researched cyclists’ eye movements by introducing fixation duration; Although many successes of eye tracking applications validate the feasibility in human training, it is seldom to see applying eye tracking technology to marine operation training [20], much less researches on analyzing and evaluating eye behavior during the training.

It is therefore believed that using eye tracker for marine operation training to study human error is reasonable and feasible. The present research is a pilot study with an aim to improve training quality of marine operation through the analysis of eye behaviors of marine operator in simulator. Considering the complexity of marine operation and the operation differences between experts and novices, how to efficiently evaluate the eye behavior is challenging. To this end, we propose a group-wise comparison method for eye behavior comparison in marine training. The paper’s main contributions can be two folds:

- A scanpath similarity comparison method which allows group-wise comparisons is proposed to compare and evaluate the performance of novice;
- A complete eye behavior evaluation in real marine crane lifting operation is carried out, together with a pattern extraction by experts’ operation.

## Related Work

### Eye movement data type analysis

During years of researches on eye-tracking area, researchers found that there are many different eye movement types which share different eye behaviors, and according to the characteristics of these eye movement, they can be briefly separated into gaze, fixation, saccade, pupil data, blink and scanpath. Analysis and evaluation of eye tracking data has been a hot topic in recent years. They can be separated simply into performance assessment, fatigue, cognitive load, risk perception, experience and expertise. In our study of marine training in this paper, it belongs to the issue of experience and expertise. 

Experience and expertise related issue is a commonly discussed topic. It is believed that operators’ experience is important, but it is abstract and hard to measure. The eye-tracking technique offers a suitable method to measure and evaluate experience of operators. Currently, there are only a few researches of eye behavior for marine operations. For example, Atik & Arslan [21] captured eye movement data from ship officers in simulation exercises and proposed a method for assessing competency between novice and expert operators in navigation training; Hareide & Ostnes [22] collected eye tracking data from both simulator and field studies for analysis and presented a recommended visual scan path for the maritime navigators. In contrast, there are many studies about experience and expertise issue using eye trackers regarding in automobile and aviation areas. Konstantopoulos et al. [23] and Paeglis et al. [13] studied the experience and expertise of drivers considering mainly on fixation related data such as mean fixation duration, mean fixation number, total fixation percentage on AOIs (area of interest), fixation percentage on specific AOIs. In this topic, scanpath comparison method is also utilized to distinguish between novice and expert. Scanpath is considered another option for eye behavior analysis. For example, Bellenkes et al. [24], Kasarskis et al. [3], Schriver et al. [25], Sullivan et al. [26] compared the novice and expert pilots by analyzing fixation related data and scanpath. Based on existing researches, it is agreeable to introduce fixation related data, such as mean fixation number, duration and fixation percentage, and scanpath to study experience and expertise related issue.

### Scanpath comparison method

‘Scanpath’, which was first mentioned by Noton & Stark [27], can also refer to a ‘scan pattern’, ‘scan sequence’ or ‘gaze sequence’, and is an eye movement sequence that consists of fixations and saccades.

There are several methods for evaluating scanpaths depending on the user’s research emphasis. The Levenshtein distance method, for example, is one useful metric to indicate spatial difference of scanpaths. It converts sequence of points of fixation into a string and measures the minimum number of operations needed to transform the string into the other [28]. Another common method is the attention map, which calculates on the basis of a set of points (raw data sample or fixations) with no order. Theoretically, it is the superposition of a Gaussian function centered on these points. The result is a Gaussian landscape, or a ‘heat map’ if represented in color. Since each scanpath can form its own attention map, it is possible to compare scanpaths by overlapping pairwise or group-wise attention maps. Comparing the scanpaths with attention map was first described by Pomplun et al. [29] when they researched disambiguating complex visual information. The method was popularized by Wooding [30] who used the attention map method to examine the large number of eye-movement traces and discussed their application to the quantification of trace similarities. 

To date, there have been a large number of methods to calculate the similarity between part-wise and group-wise attention maps. For instance, Wooding [31] designed the simple subtraction method to quantify the similarities between part-wise attention maps. With a critical threshold value of d, the sum coverage would be the similarity. Ouerhani et al. [32] proposed a correlation coefficient to calculate the attention map similarity and also publicized the algorithm that could transform the fixation points to a human attention map. The study showed the relationship between fixation distribution patterns and common visual search behaviors. Besides that, Rajashekar et al. [33] and Nyström et al. [34] raised the idea of KullbackLeibler divergence to compare scanpath similarities; Caldara & Miellet [35] proposed a new method named the iMap statistical fixation mapping of eye movement data and comparison of scanpaths; Grindinger et al. [36] proposed a method on group-wise scanpath comparison based on expert scanpath and can be visualized by heat map. Inspired by Grindinger’s method [36], a new scanpath comparison method is proposed that can effectively evaluate novice performance in group-wise. It is introduced in detail in Section 3.

## Methodology

According to Section 2.1, several fixation metrics including fixation percentage, mean fixation number and duration are thus applied for analysis of eye behavior in marine operation training. In addition, a new scanpath similarity comparison method inspired by Grindinger’s method [36] is proposed to fit marine operation training. The goal of this method is to evaluate the performance of novice operators based on the expert operators’ eye-movement data.

**Figure 1:**
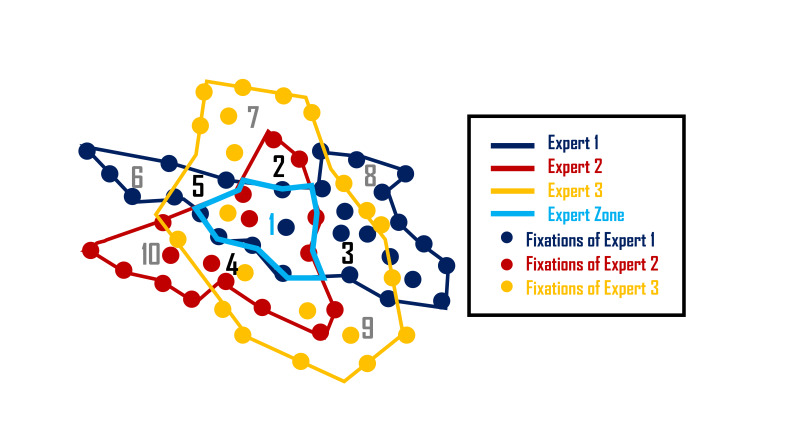
Example of how expert zone established in three-expert condition.

The expert zone, can be easily understood as the intersection areas of two or more expert operators’ fixations. Basically, each expert’s fixations are first clustered with clear boundary according to its spatial distribution. Then the intersected area of two or more expert fixation boundaries is extracted and named the expert zone. Figure 1 shows a schematic example of the establishment of expert zone in three-expert condition. The boundary of each expert’s fixations may be depicted as a complex polygon. Here region 1 with blue boundary is the expert zone. Besides expert zone region 1 in Figure 1, it is also emphasized that the two-expert areas from regions 2 to 5; the single expert area from region 6 to region 10. These areas are also critical when evaluating novice operator performance.

Once experts’ fixated boundary has been determined, the distribution of novices’ fixations, i.e., which regions the fixations belong to, can be identified. In this way, the percentage of novices’ fixations in the ‘Expert zone’ and expert fixated areas can be calculated for eye behavior evaluation of the novice operators. To study this issue in a general way, when there are *N* experts as reference to evaluate the performance of novice operators, each fixation from novice in the expert zone is given 1 point, each fixation in n-expert (*n<N*) intersected areas but not in expert zone is given *n/N* points, then summarizing all the points, dividing by total fixations of novices, their performance score is calculated. Table 1 summarizes points in different regions.

**Table 1:** Points in different regions.

Region	Point
In expert zone	1
In n-expert (*n<N*) intersected areas but not in expert zone	*n/N*
Otherwise	0

Based on the explanation above, the score formula for novice operators is proposed:


(1) $$Final\_Score=\sum_{i=1}^{m}\sum_{n=1}^{N}\frac{n}{N}\times \omega_{i}\times \frac{P_{ni}}{P}$$

where *m* is the total AOI number; *N* is the total expert number; *n* is a positive integer always less than or equal to *N*; *P_ni_* are fixations in *n*-expert intersected areas but not in the expert zone of a novice in AOI_i_; *P* are total fixations of a novice in AOI_i_; ω_i_ are weight number in AOI_i_.

Weight number is considered in  equation 1, since there are different priorities for fixations in different AOIs. The fixations in critical AOIs are more valuable than those in other AOIs. In such a way, one can evaluate each novice operator’s performance in all defined AOIs. Furthermore, it is possible to extract critical information from the analyzed data to figure out the pattern modes of eye behavior in marine operation training.

## Experiment

A marine crane-lifting experiment in marine operation simulator was conducted using eye trackers to study the difference in eye behavior between expert and novice marine operators using the aforementioned methods.

### Goal

For this marine crane-lifting experiment, there are two main goals. The first is to study the visual behaviors of operators and conclude the rules behind them by comparing similar and dissimilar features between novice and expert operators. The second is using the aforementioned evaluation method to prove its feasibility in real cases. The marine crane lifting experiment is an example to study the implementation of this methodology.

### Participants

Ten male marine crane operators participated in this study. All of them had experience in marine operation and were divided into two groups. The novice group had six participants with experience ranging from 4 to 6 years (m = 5.16, SD = 0.98). The expert group had four participants who had experience ranging from 8 to 10 years (m = 9.00, SD = 1.15). They had the same schedules for this experiment except that some participants from the novice group did some pre-practice in the marine simulator. The aim is to let participants get familiar with the devices. Since the pre-practice scenario is totally different from the experiment, it will not affect the experimental result. Considering the incompleteness of data samples for some participants, the main experimental data is composed of the data from four novice participants and two expert participants.

### Apparatus

To measure the eye movements of the participants, we used Tobii Pro 2 glasses to collect data. To analyze the data, we mainly utilized Tobii studio analysis software and MATLAB.

### Scenario

An offshore construction vessel with a knuckle boom crane is used in the experiment. The scenario is to lift a suction anchor (weight 80 tons, height 20 meters, diameter 5.3 meters) from the deck of the vessel to the seabed at a depth of 100m. The whole operation took each participant around 30 minutes.

### Questionnaire

After finishing the experiment, every participant operator was required to fill in a questionnaire to give feedback on the finished operation. This questionnaire is of importance to define the size and shape of the AOIs, and also for deciding the weight number proposed in the evaluation method.

The questionnaire includes the following questions:

- Where are the crucial areas during the experiment?
- Where is your visual focus when the load is in the air?

### AOI definition

**Figure 2:**
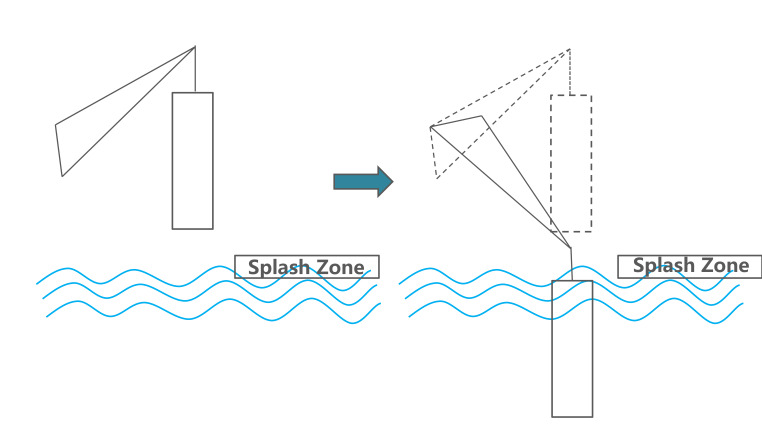
Target stage one: load descending from air to total immerse.

**Figure 3:**
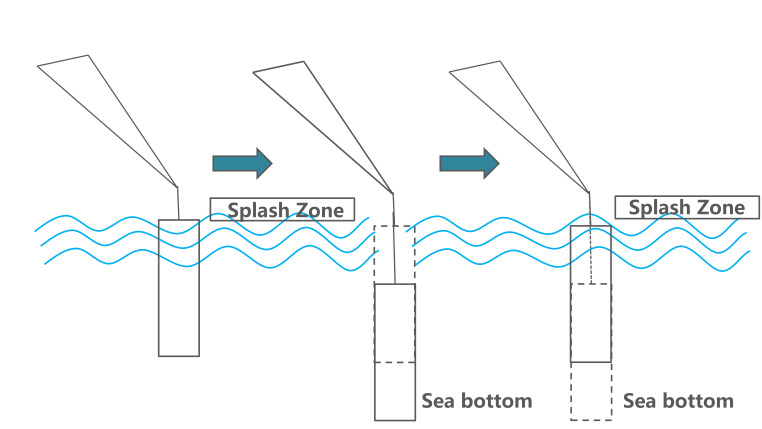
Target stage two: load descending to seabed and back to water surface.

In this experiment, to analyze and study with high efficiency, two segments were selected. Target stage one can be described as ‘descending the load until total immersion’, as shown in Figure 2; target stage two can be described as ‘total underwater period of the load’, as illustrated in Figure 3.

Then the attention maps of all participant operators using the software Tobii Pro Lab are plotted, and the main focus was given to the attention map of expert. After superposing attention maps of all experts in the target stages, the peak of this Gaussian landscape model was cut manually by referring to the questionnaires done by the participants to decide the AOIs. To decide where to cut the peak of the Gaussian model, the questionnaires written by the participant operators were taken as the reference for deciding the AOIs. Figure 4 shows the results of AOI definition for target stage one and two, in which ‘Monitor 1’ displays the sensor data about the underwater situation, such as rope paying out and tension force; ‘Monitor 2’ is from the camera mounted on the crane-tip, which visualizes the tension of crane rope during operation; ‘Swing of load and sinking process’ covers the potential area with high risk of collision before it totally submerges into the water; and ‘Crane tip’ is the stress concentration area that determines operation safety in this experiment.

Figure 4AOI definition for (a) target stage one, and (b) target stage two.

**Figure:**
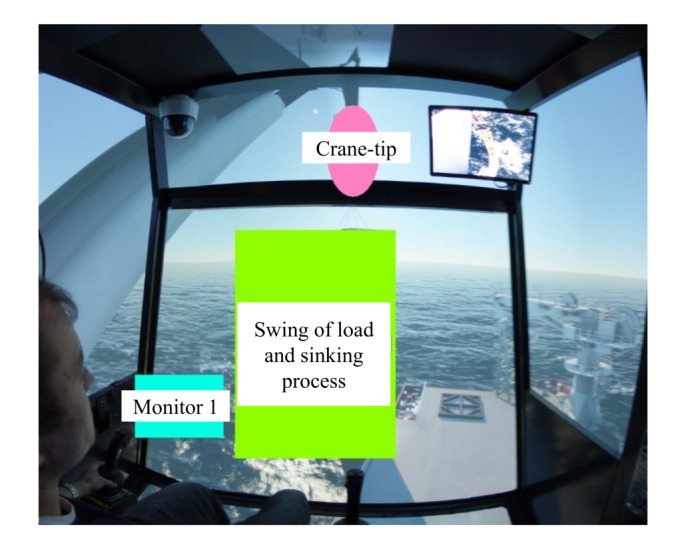
(a)

**Figure:**
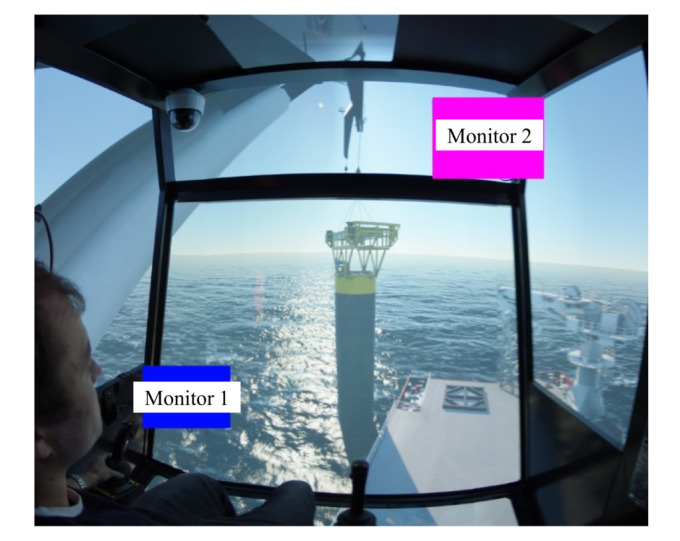
(b)

## Results

To study the experience and expertise, following by related works as mentioned above, fixation related data and scanpath are mainly measured and analyzed. For fixation related data, mean fixation number, total fixation percentage on AOIs and fixation percentage on specific AOIs are introduced.

### Mean fixation number and total fixation percentage on AOIs

Mean fixation number is the parameter that can indicate how many fixation times one has over a defined period of time. The total fixation percentage on defined AOIs is also introduced, which is the parameter that indicates the percentage of ‘effective’ fixation through the whole operation.

Figure 5Mean fixation number and total fixation percentage on AOIs at (a) target stage one, and (b) target stage two.

**Figure:**
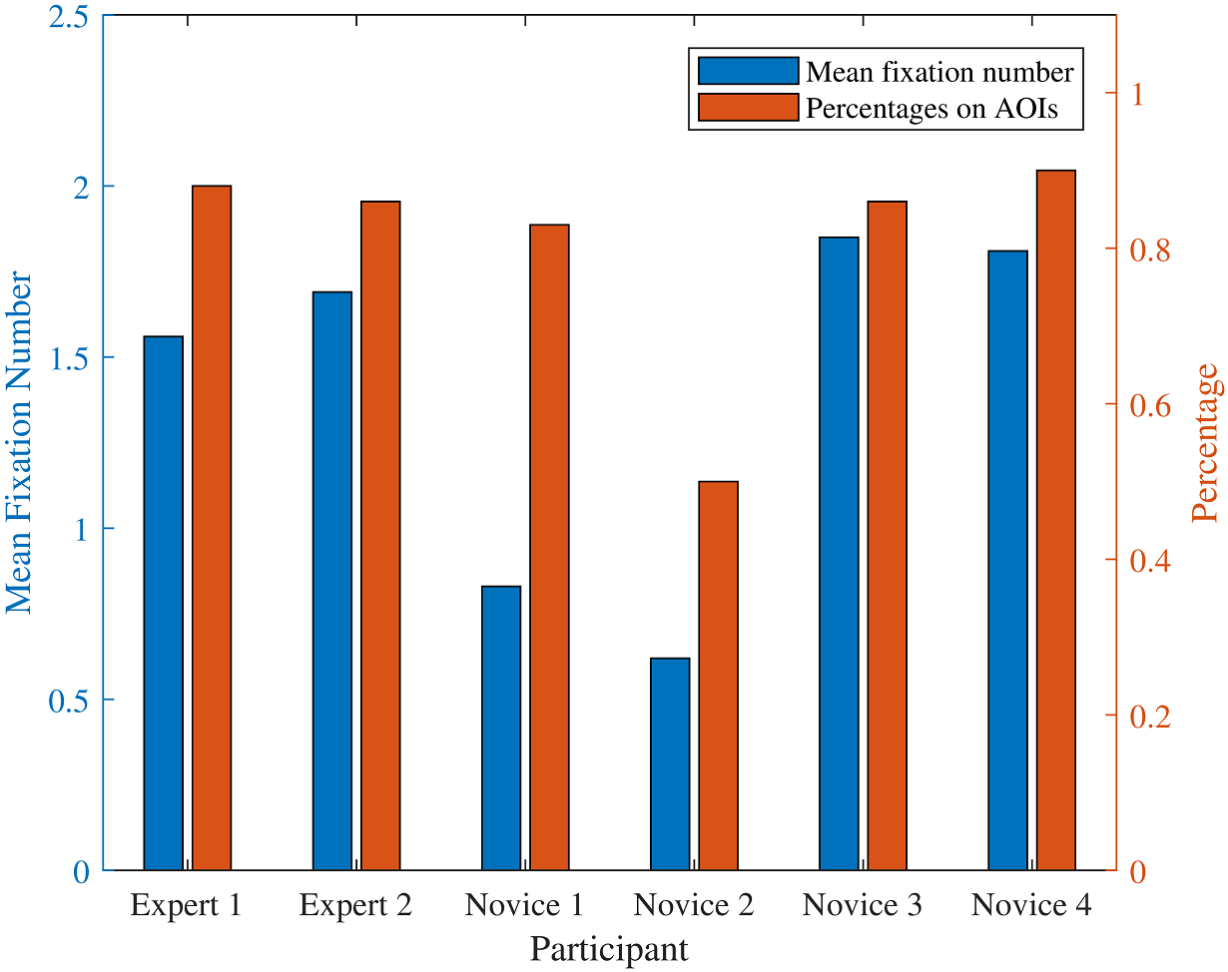
(a)

**Figure:**
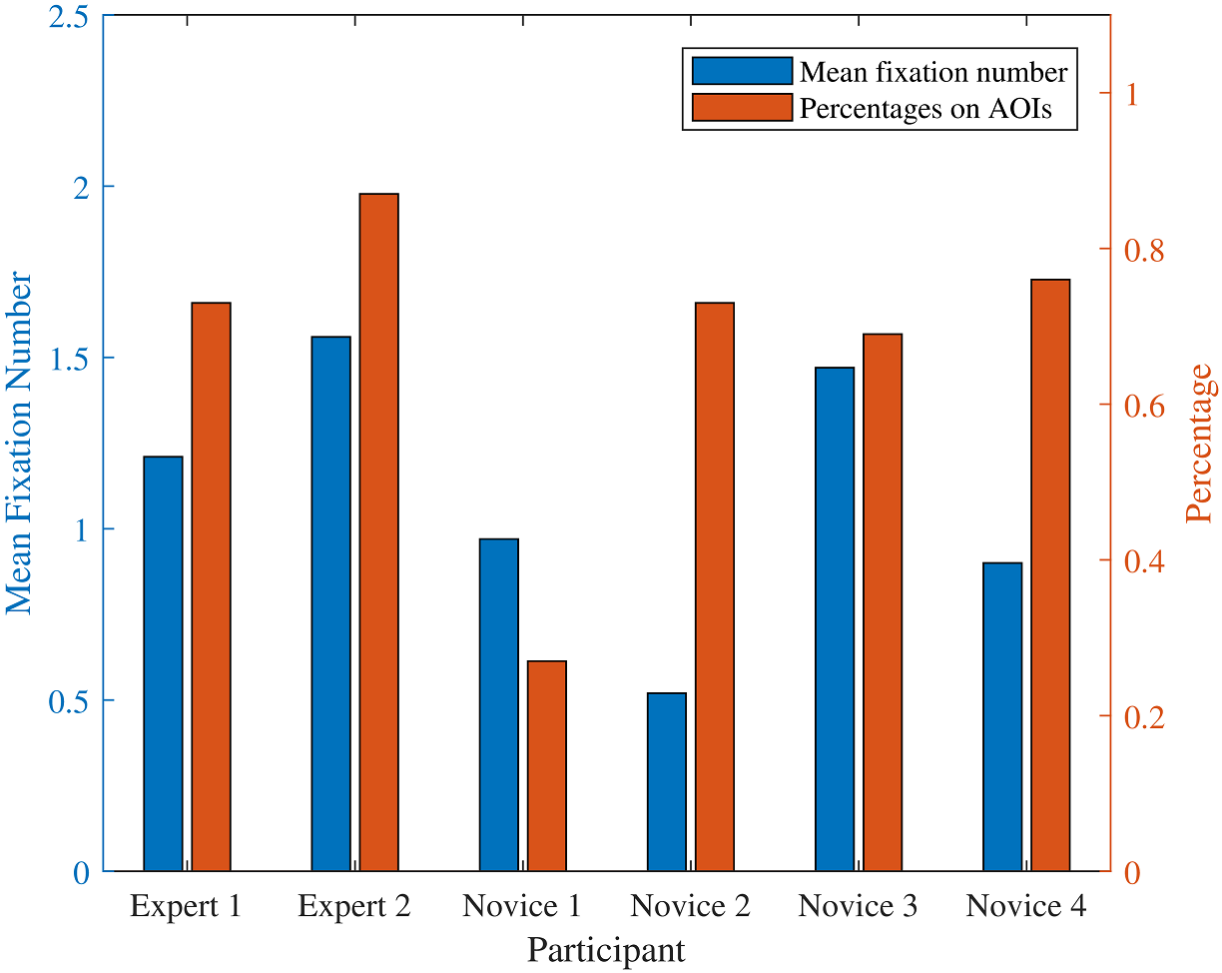
(b)

For target stage one, Figure 5a shows two of the novice operators fixated most among all participants with mean fixation number 1.85 and 1.81, respectively; the rest two novice operators have a lower number of 0.83 and 0.62, respectively. The expert operators have the mean fixation number with 1.69 and 1.56 where these numbers are in the middle level among all participants. For fixation percentage on AOIs, all of the participants performed high percentage on AOIs ranging from 83% to 90% except for ‘Novice 2’ with only 50% fixation percentage. Expert operators and novice operators share similar percentage in this stage. 

For target stage two, Figure 5b shows that both the experts obtained high mean fixation number. The novices except ‘Novice 3’ obtained relatively low mean fixation number ranging from 0.52 to 0.97. In addition, both the experts and the novices except ‘Novice 1’ performed high fixation percentage on defined AOIs. 

In these two stages, it can be seen that although the experts do not always have the highest mean fixation number and fixation percentage compared to novice operators, they have a relatively stable eye movement in terms of mean fixation number and fixation percentage.

### Fixation percentage on specific AOIs

In this part, fixation percentages on specific AOIs is calculated and analyzed. Different from the total fixation percentage mentioned above, studying the fixation percentage on specific AOIs can better indicate how the operator allocates their spatial fixation on each AOI.

Figure 6a shows the fixation percentage in each AOI for target stage one. It can be seen that ‘Swing of load and sinking process area’ has the highest fixation percentage for all operators. Even there are three novices obtained higher fixation percentage than that of the experts. This is reasonable because from the questionnaire, all participants wrote that the swing of load is quite worth fixating in this target stage. All participants except ‘Novice 1’ fixated on ‘Monitor 1’ area in this stage and ‘Expert 1’ obtained the highest fixation percentage up to 35%. Four of the participants fixated on ‘Crane-tip’ area but with a relatively low fixation percentage. From Figure 6a, the experts tend to allocate fixations among the three AOIs; while the novices pay less attention on ‘Monitor 1’ and ‘Crane-tip’ but fixate more on ‘Swing of load’ area.

Figure 6b illustrates the result of fixation percentage on ‘Monitor 1’ and ‘Monitor 2’ in target stage two. All participants put more effort on ‘Monitor 1’ and only ‘Novice 2’ allocates fixations evenly between the two AOIs. In fact, after the load fully sinking into the sea, there are no target on the sea surface. The two AOIs play important roles in this case, where ‘Monitor 1’ displays the sensor data about the underwater situation and ‘Monitor 2’ visualizes the tension of crane rope during operation. Therefore, it is reasonable to fixate most of time on ‘Monitor 1’ and only keep necessary focus on ‘Monitor 2’ as well.

Figure 6Fixation percentage on AOIs at (a) target stage one, and (b) target stage two.

**Figure:**
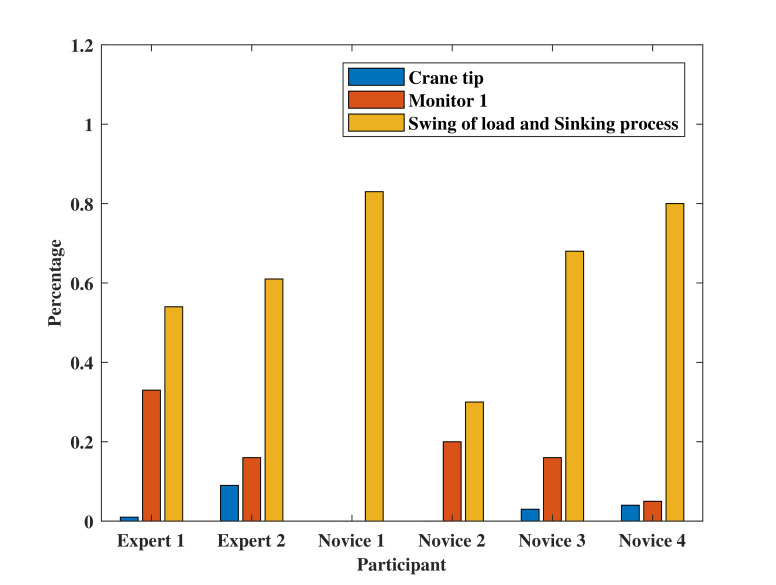
(a)

**Figure:**
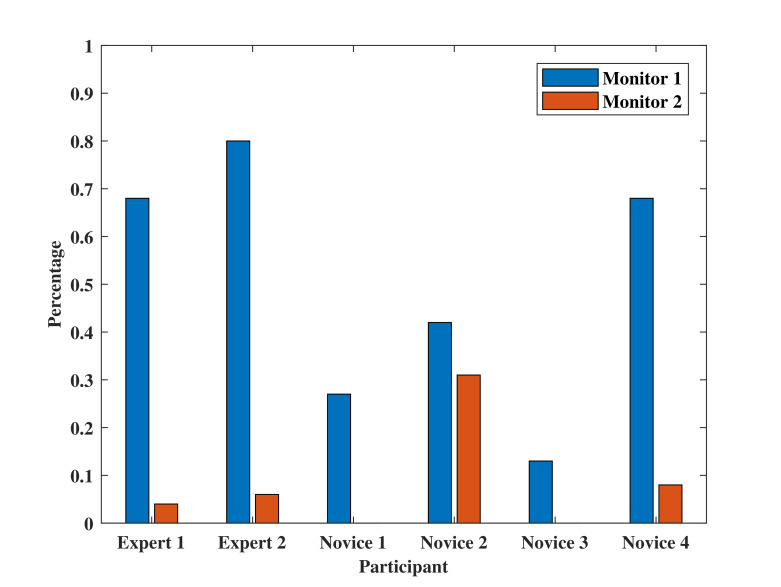
(b)

### Mean fixation duration and its standard deviation

In this section, mean fixation duration with its standard deviation of participant operators in target stages are measured and visualized. Normal distribution of the mean fixation duration is used to better understand the eye behaviors differences between experts and novices.

Figure 7a depicts that the mean fixation duration ranges from 226 ms to 471 ms in target stage one. The experts tend to perform a longer fixation duration with a wide standard deviation. The novices, however, have totally different distributions. In particular, ‘Novice 1’ and ‘Novice 3’ perform a shorter fixation duration with a narrow standard deviation, which shows different pattern compared to that of the experts.

The mean fixation duration for target stage two is shown in Figure 7b, ‘Novice 1’ and ‘Expert 1’ perform the shortest and longest mean fixation duration for 340 ms to 838 ms in this stage, respectively. The average duration is longer compared to that in target stage one. The reason is that in target stage two, the task is relatively easy, and it is unnecessary for the participants to switch between the two AOIs. The standard deviation ranges from 388 ms to 1816 ms, which is also larger than target stage one. It is hard to distinguish the experts and the novices from Figure 7b, because of the huge difference in standard deviation between each participant in this stage.

Figure 7Mean fixation duration with standard deviation at (a) target stage one, and (b) target stage two.

**Figure:**
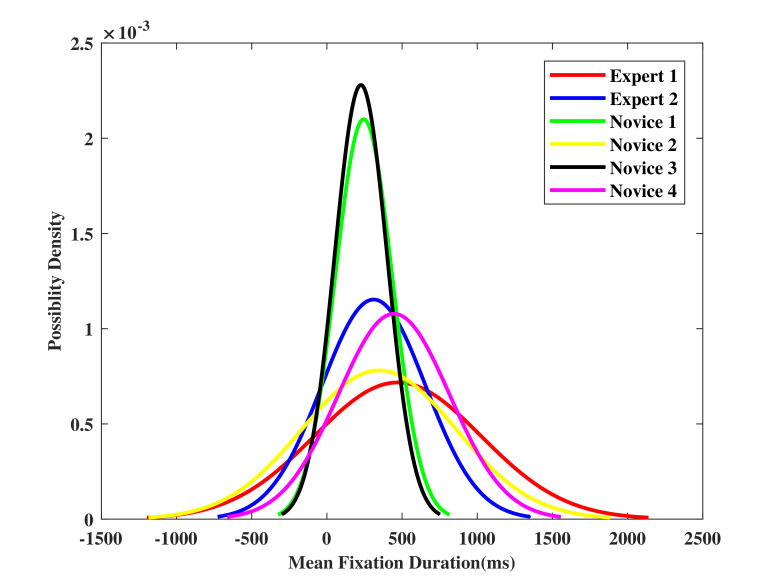
(a)

**Figure:**
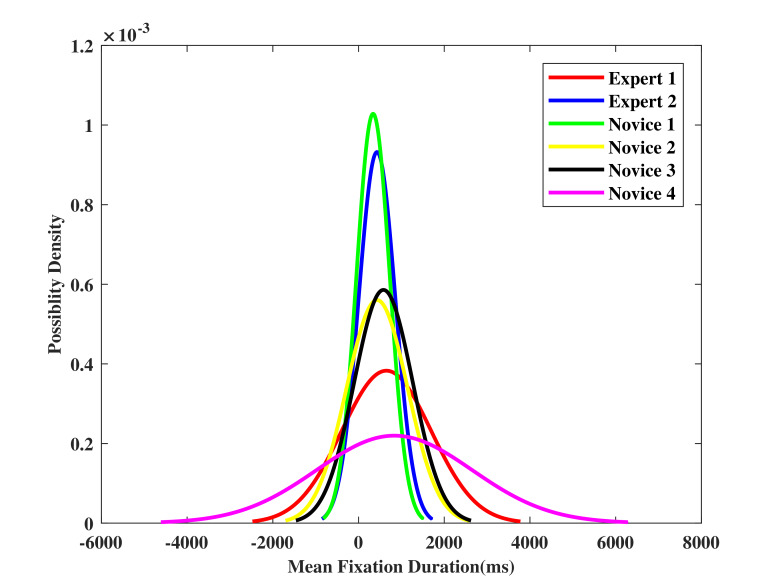
(b)

### Scanpath analysis

In this part, scanpath analysis is conducted. Considering full time period scanpath may cause complex visualization data, we use ‘critical area’ to simplify the scanpath from both spatial and temporal aspects. The ‘critical area’ is based on the questionnaires. As a result, ‘Swing of load’ is selected as the ‘critical area’ for target stage one, while for target stage two, the ‘critical area’ is the one-minute time period when the load reach the seabed.

Figure 8Scanpath for (a) experts and (b) novices in target stage one.

**Figure:**
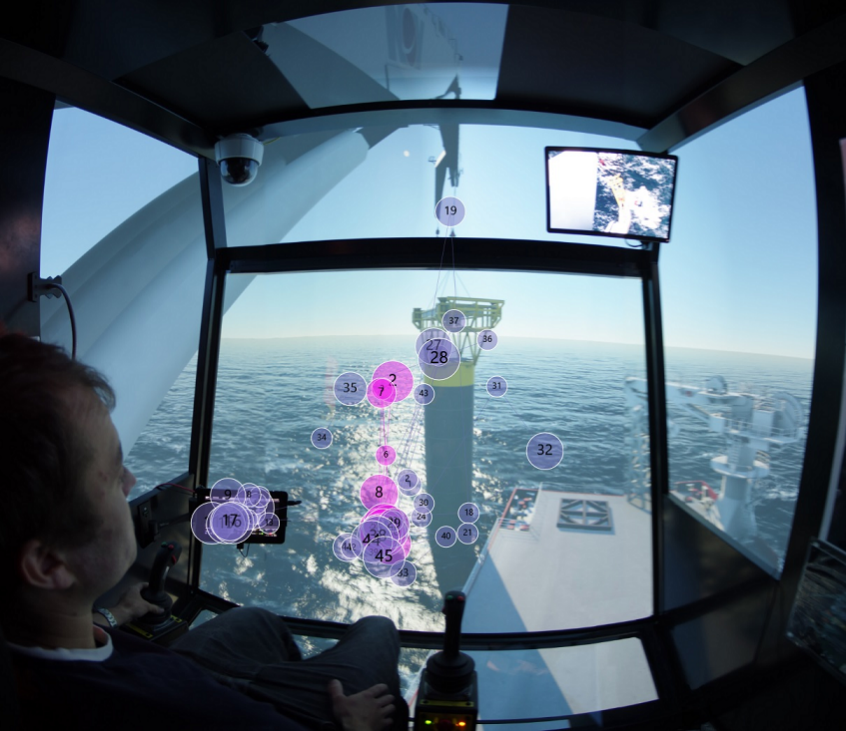
(a)

**Figure:**
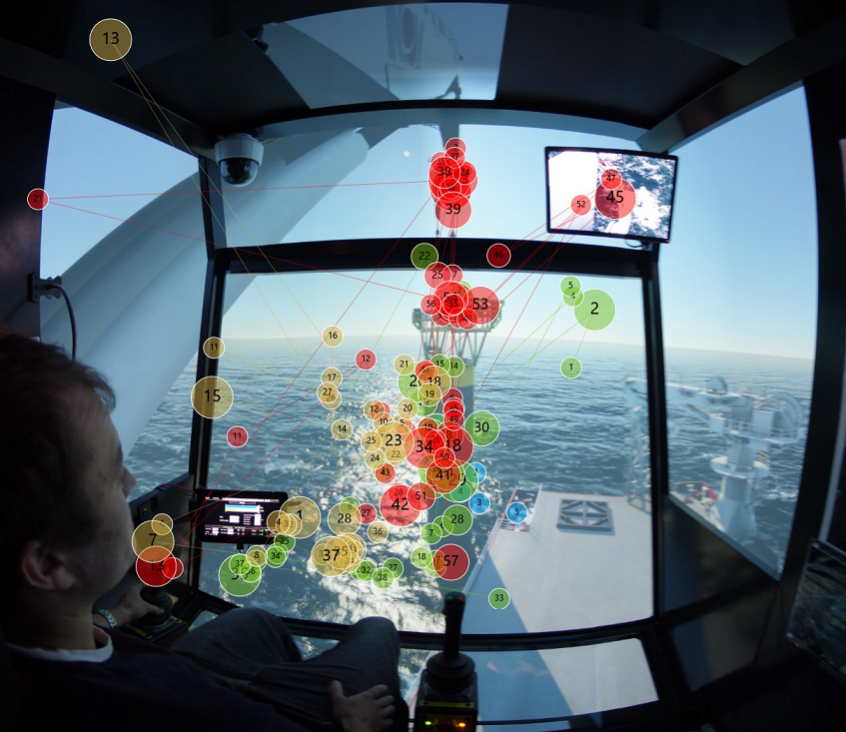
(b)

From Figure 8a, by combining with the ‘critical area’ in target stage one, it would be seen that experts fixate mostly in ‘Swing of load’ and move with bottom of the load as it descends. In contrast to experts’ scanpaths, Figure 8b shows that some novices tend to fixate more on ‘Crane tip’ while some novices care more on ‘Swing of load’; the scanpaths of novices are more scattered, which implies the novices have their own visual focus and strategies.

Regarding scanpath in target stage two, Figure 9a shows that experts allocate most of their fixations between ‘Monitor 1’ and ‘Monitor 2’. It is observed that they also focus on the sinking point of the load at the center of the screen. For novices, from Figure 9b, they also fixate on the two monitors and the sinking point back and forth. However, because some novices have focused on the ‘Crane tip’ that the experts do not pay attention to in target stage two, it is considered a slight behavioral difference compared to the experts.

Figure 9Scanpath for (a) experts and (b) novices in target stage two.

**Figure:**
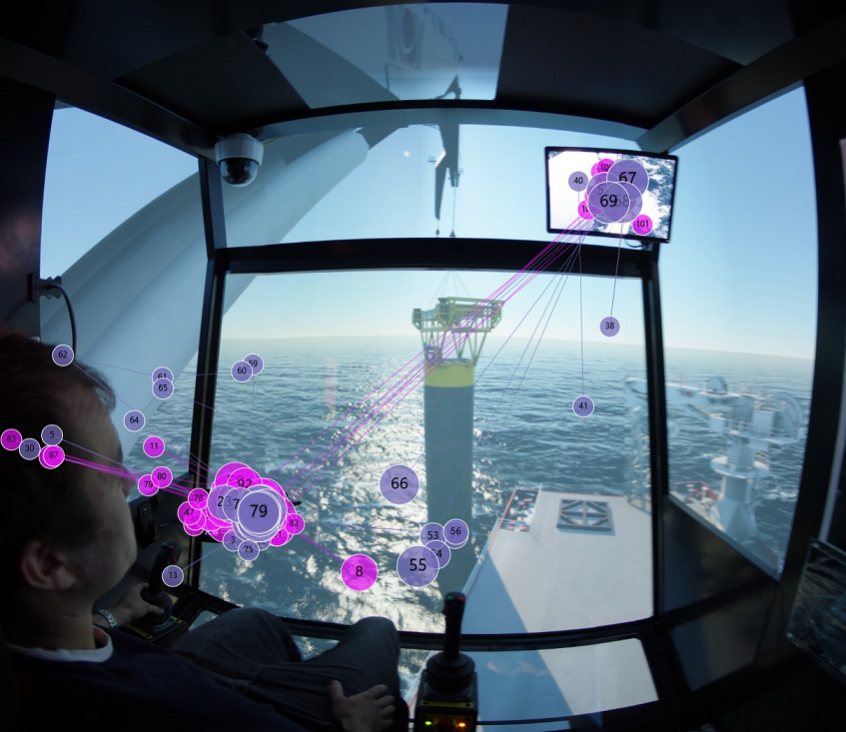
(a)

**Figure:**
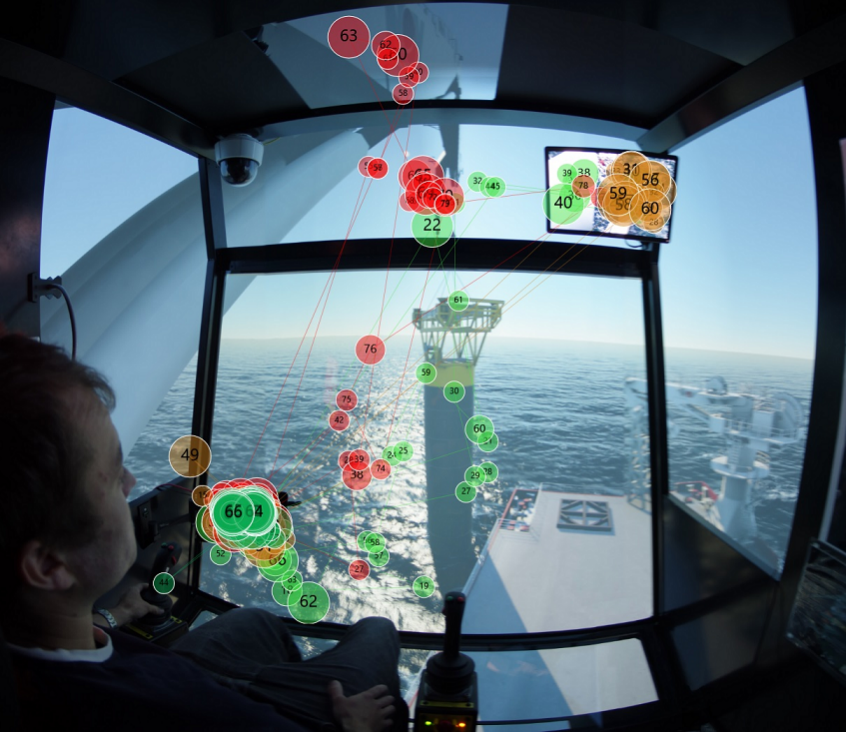
(b)

### Scanpath evaluation

The proposed evaluation method in Section 3 is used here to evaluate and compare eye behavior of the novices and experts during the training.

**Table 2:** Scores of eye behavior for novices in target stage one.

Participant	AOI	Fixation percentage			Score	
		{Expert zone}	{Expert 1}/{Expert zone}	{Expert 2}/{Expert zone}	In AOI	Total
Novice 1	Crane tip	0.58	0.07	0.31	0.15	0.77
	Swing of load	0.74	0.16	0.05	0.59	
	Monitor 1	0	0.51	0	0.03	
Novice 2	Crane tip	0	0	0	0	0.65
	Swing of load	0.76	0.09	0.05	0.58	
	Monitor 1	0.22	0.74	0.18	0.07	
Novice 3	Crane tip	0	0	0	0	0.66
	Swing of load	0.92	0.05	0.01	0.66	
	Monitor 1	0	0	0	0	
Novice 4	Crane tip	0	0	0	0	0.67
	Swing of load	0.71	0.18	0.15	0.61	
	Monitor 1	0.16	0.67	0.12	0.06	

The fixation points within the defined AOIs are considered. In order to establish expert zone, the boundary of each expert’s fixation around the defined AOIs is calculated, followed by an exclusion of non-overlapping area. Figure 10 shows the expert zones for the two of the target stages. It is obvious that the generated expert zones are irregular polygons, and smaller than the areas of the defined AOIs.

Figure 10Defined ‘Expert zone’ in (a) target stage one, and (b) target stage two.

**Figure:**
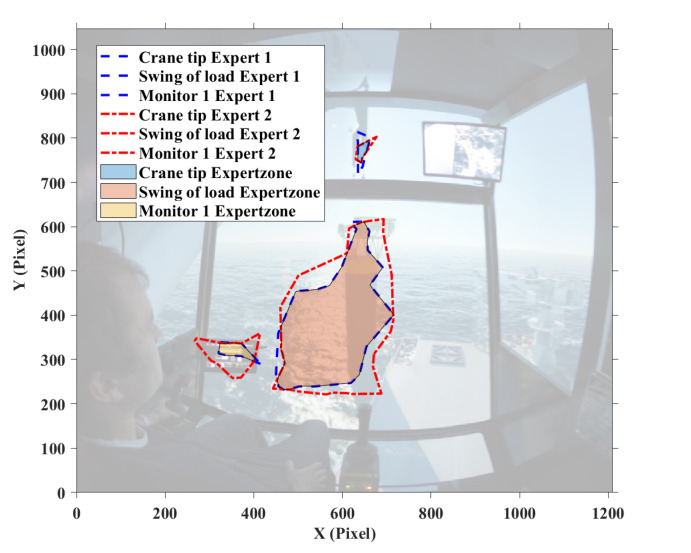
(a)

**Figure:**
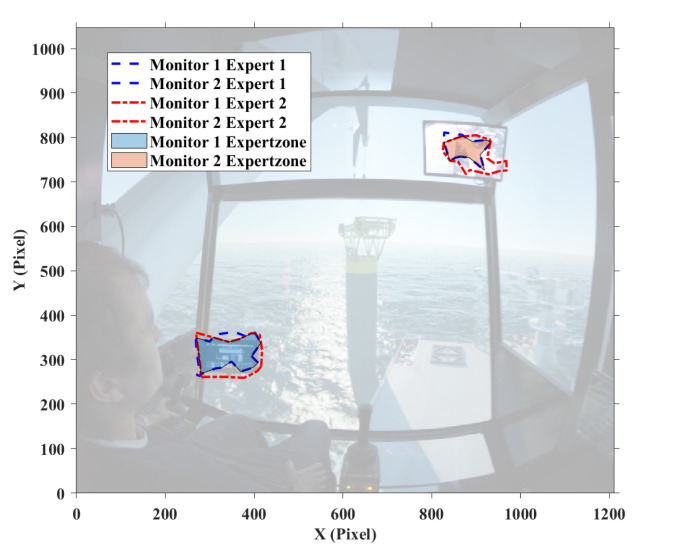
(b)

Next, as far as the distribution of novices’ fixations is known, the percentage of their fixations in ‘Expert zone’ are calculated. In addition, the fixations located within the boundary of a single expert’s fixations but outside the ‘Expert zone’ are also evaluated. All of the fixations are associated with different weight numbers that are determined on the basis of the questionnaires by participants. In this experiment, the weight numbers for ‘Swing of load’, ‘Crane tip’ and ‘Monitor 1’ are set to 0.7, 0.2 and 0.1, respectively, in target stage one; the weight numbers for ‘Monitor 1’ and ‘Monitor 2’ are assigned to 0.7 and 0.3 in target stage two. Eventually, according to Equation (1), score of novice operators can be calculated.

**Table 3:** Scores of eye behavior for novices in target stage two

Participant	AOI	Fixation percentage			Score	
		{Expert zone}	{Expert 1}/{Expert zone}	{Expert 2}/{Expert zone}	In AOI	Total
Novice 1	Monitor 1	0.21	0.79	0.75	0.69	0.88
	Monitor 2	0.52	0.15	0.11	0.19	
Novice 2	Monitor 1	0.39	0.60	0.60	0.69	0.69
	Monitor 2	0	0	0	0	
Novice 3	Monitor 1	0.09	0.64	0.29	0.39	0.54
	Monitor 2	0.06	0.39	0.46	0.15	
Novice 4	Monitor 1	0.28	0.28	0.64	0.66	0.87
	Monitor 2	0.36	0.36	0.18	0.21	

Table 2 lists the score of the novices’ eye behavior in target stage one. ‘Novice 1’ obtains the highest score of 0.77 and the rest novices have a similar result around 0.66. It is revealed from Table 2 that ‘Novice 1’ has fixations on ‘Crane tip’ and thus obtains extra score. This indicates the eye behavior of ‘Novice 1’ is more similar to that of experts shown in Figure 8a. In addition, it is noted that the majority of the scores comes from the ‘Swing of load’ AOI. From the score distribution in ‘Expert zone’, all novices have relatively high percentage of fixation on this AOI with the highest weight number of 0.7 in this stage. This implies setting the high weight number on this AOI is reasonable.

The scores for novices’ eye behavior in target stage two is listed in Table 3. There is large variation among them that the highest score is up to 0.88 while the lowest one is only 0.54. It is interesting to see that the majority of their scores derive from ‘Monitor 1’ area; the fixation percentage outside the expert zone of ‘Monitor 1’ is high and contribute most of the scores.

### Discussion

This section discusses the relationship between operation time, experience and task difficulty, summarizes eye behavior statistics for both experts and novices, extracts scan modes of experts in the two target stages, and prospects how the proposed method could be applied in training programs.

 From Figure 4, we can see that target stage one is much more difficult compared to target stage two, since more areas need to be focused during the operation. The expert group performed more efficiently than the novice group (178±16s vs. 204±41s) in this stage. While in target stage two, both groups have a similar operation time (986±136s vs. 1020 ± 95s). The result implies the operation time depends not only on the degree of familiarity of the device but also on the difficulty level of the task. 

The expert group in the experiment have a stable scan mode. Regarding the mean fixation number in Figure 5, one can see from the plot of the target stages, experts do not always have the highest mean fixation number in every stage; whereas they are moderately stable and high in the two target stages. In addition, experts have relatively high and stable total fixation percentage on the AOIs of the two target stages, as well as similar fixation percentages on specific AOIs. This indicates the correctness of the defined AOIs in Figure 4. When considering mean fixation duration and its standard derivation, experts do not have obvious features that can separate from novices. Different operators have certain strategies, which leads to similar mean fixation duration and standard derivation features in the two target stages. However, in terms of novices, they share an unstable mean fixation number distribution in target stages and so do the total fixation percentage on the AOIs and fixation percentages on specific AOIs.

Regarding the scanpaths of all the participants, we summarize the scan modes of experts in the two target stages. The scanpaths of experts are distinguished from these of novices in that experts fixate more frequently on the ‘Swing of load’, ‘Monitor 1’ and ‘Monitor 2’ areas. More specifically, for target stage one, experts conduct a wider range of observations, that is, to scan left and right, to up and down in ‘Swing of load’ AOI to ensure the safety of the load, as shown in Figure 11a; for target stage two, Figure 11b depicts that experts switch focus between ‘Monitor 1’ and ‘Monitor 2’ areas frequently, and occasionally keep eyes on the water surface to observe the rope tension.

Figure 11Extracted scanpath mode in (a) target stage one, and (b) target stage two.

**Figure:**
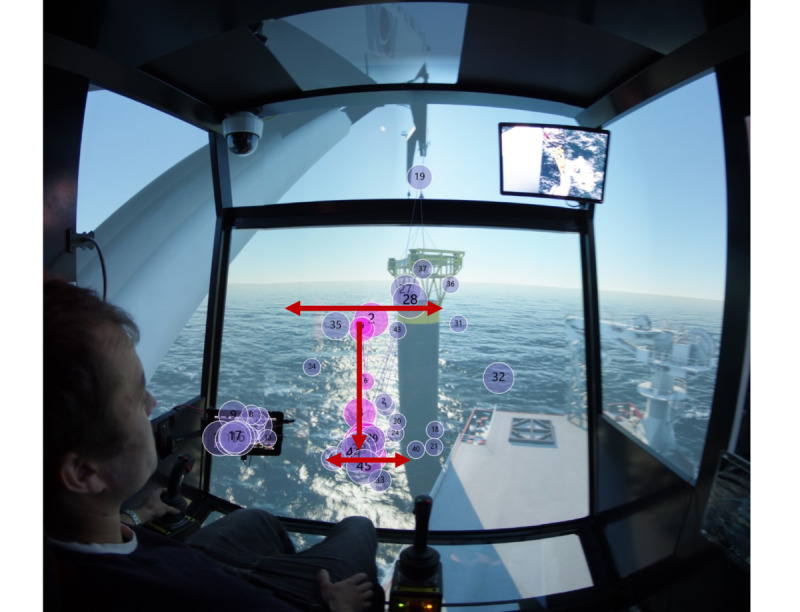
(a)

**Figure:**
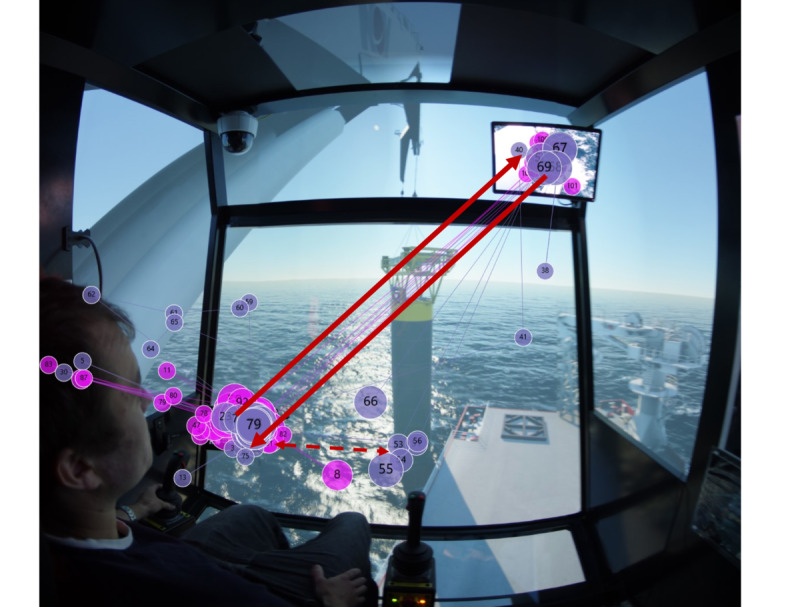
(b)

The eye behavior for novices in the experiment varies from person to person. Some novices, such as ‘Novice 4’, can perform similar eye behavior such as long mean fixation duration and standard deviation to that of ‘Expert 1’ in the two target stages in Figure 7; whereas some novices like ‘Novice 3’, have obvious difference compared to ‘Expert 1’. From Table 2 and Table 3, it is concluded that using the statistical metrics on eye tracking data indeed distinguishes the eye behavior to some extent, even though different participants have different scan strategies during marine operation training.

The proposed eye behavior evaluation method is also promising for improving the training program of marine operations in two aspects. First, the AOIs, the ‘critical area’ and the extracted scan patterns could be emphasized in the briefing phase, to guild the trainees to grasp the key skills of the operation. Second, in debriefing phase, a comprehensive analysis of the evaluation results, especially the fixation percentage, the mean fixation number and duration, and scanpath, will be served as the basis to help the trainees to reflect on why the deviations happen and how to avoid them. As a result, the method will accelerate the training progress without losing quality.

## Conclusion

This paper presents a new analysis approach for evaluating situation awareness in marine operation training. A term ‘Expert zone’ representing the similarity of fixations among experts in a form of overlapping AOI is introduced. According to the questionnaire from experts, the AOIs and their weight numbers are recognized. A scanpath evaluation for novices is thus established to quantify how similar the eye behaviors of novices compared to these of experts. 

A pilot study of crane lifting experiment was carried out in simulator. Two target stages of the operation were selected. A comprehensive analysis on different eye tracking metrics together with the scanpath evaluation is conducted between both novices and experts. The results show the effectiveness of the proposed method in assessing eye behavior for marine operation training.

For future work, efforts will be put into (1) involving more participants in the same experiment and applying statistical hypothesis tests for better understanding of eye behaviors between novices and experts in marine operation training; (2) refining the scanpath comparison method by integrating more eye tracking metrics; and (3) conducting other types of marine operation experiments to verify the scope of adaptation of the proposed method.

### Ethics and Conflict of Interest

The authors declare that the contents of the article are in agreement with the ethics described in http://biblio.unibe.ch/portale/elibrary/BOP/jemr/ethics.html and that there is no conflict of interest regarding the publication of this paper. 

### Acknowledgements

The research is supported in part by a grant from RFF Midt-Norge “On-Board Augmented Simulation” (Project nr: 283856), and in part by a grant from the Research Based Innovation “SFI Marine Operation in Virtual Environment (SFI-MOVE)” (Project nr: 237929) in Norway. 
